# Use of ranitidine is associated with infections in newborns hospitalized in a neonatal intensive care unit: a cohort study

**DOI:** 10.1186/s12879-017-2482-x

**Published:** 2017-05-30

**Authors:** Ruth N. S. Santana, Victor S. Santos, Ruy F. Ribeiro-Júnior, Marina S. Freire, Maria A. S. Menezes, Rosana Cipolotti, Ricardo Q. Gurgel

**Affiliations:** 1 Department of Medicine, Federal University of Sergipe, R. Cláudio Batista, s/n - Cidade Nova, Aracaju, 49060-108 Brazil; 2 Postgraduate Program in Health Sciences, Federal University of Sergipe, R. Cláudio Batista, s/n - Cidade Nova, Aracaju, 49060-108 Brazil

**Keywords:** Ranitidine, Neonates, Infection, Necrotising enterocolitis, Mortality

## Abstract

**Background:**

The inhibition of gastric acid secretion with ranitidine is frequently prescribed off-label to newborns admitted to neonatal intensive care units (NICU). Some studies show that the use of inhibitors of gastric acid secretion (IGAS) may predispose to infections and necrotising enterocolitis (NEC), but there are few data to confirm this association. This study aimed to compare the rates of neonatal infections and NEC among preterm infants (<37 weeks gestation) hospitalised in a NICU exposed or not to treatment with ranitidine.

**Methods:**

A retrospective cohort study was conducted with all consecutive preterm newborns admitted to a NICU between August-2014 and October-2015. The rates of infection, NEC, and death of newborns exposed or not to ranitidine were recorded.

**Results:**

A total of 300 newborns were enrolled, of which 115 had received ranitidine and 185 had not. The two groups were similar with regard to the main demographic and clinical characteristics. Forty-eight (41.7%) of the 115 infants exposed to ranitidine and 49 (26.5%) of the 185 infants not exposed were infected (RR = 1.6, 95%CI 1.1–2.2, *p* = 0.006). The late onset (>48 h) blood culture positive infection rate was higher in the group exposed to ranitidine than in the untreated group (13.0% vs. 3.8%, *p* = 0.001). There was no significant association between the use of ranitidine and NEC (Bell stage >II) (*p* = 0.36). The mortality rate risk was 4-fold higher in infants receiving ranitidine (16.5% vs. 8.6%, *p* < 0.001).

**Conclusion:**

Ranitidine use in neonates was associated with an increased risk of infections and mortality, but not with NEC.

**Electronic supplementary material:**

The online version of this article (doi:10.1186/s12879-017-2482-x) contains supplementary material, which is available to authorized users.

## Background

Infections are the major cause of mortality in preterm newborns worldwide [1, 2]. It is well established that the gastric acid secretion is an important non-immune defence barrier for infants against invading pathogens [3, 4]. Although some studies have shown that the use of inhibitors of gastric acid secretion (IGAS) may predispose to infections [5–8] and/or necrotising enterocolitis (NEC) [7, 9], few data are currently available to confirm this association [10]. In addition, most of these studies are based on industrialised countries and there is very limited information on whether these patterns also apply to low- and middle-income populations, where the burden of infection is often highest. Due to the perceived safety and efficacy in older populations, the IGAS therapy has been commonly prescribed off-label in Neonatal Intensive Care Units (NICU) [11, 12]. In this context, we compared the rates of hospital infections, mortality, and NEC between preterm newborns exposed or not to ranitidine therapy admitted to a NICU in Sergipe, Brazil.

## Methods

### Study design and populations

We performed a retrospective cohort study to compare rates of hospital infections among premature neonates hospitalised in a NICU who were exposed or not to ranitidine treatment in the *Nossa Senhora de Lourdes* Maternity (NSLM). This maternity unit is located in Aracaju, Sergipe-Brazil and it is the high and medium-risk obstetric reference unit for the state of Sergipe. In 2014, approximately 370 births occurred per month in NSLM.

All consecutive neonates with a gestational age < 37 weeks, born at NSLM and with at least five consecutive days hospitalized in the NICU, between August 2014 and October 2015, were eligible for the study. Neonates born from mothers with trans-placental infection potential (i.e. human immunodeficiency virus, syphilis, hepatitis, toxoplasmosis, rubella and cytomegalovirus), patients with congenital malformation (i.e, hydrocephalus, intestinal atresia, gastroschisis, meningoencephalocele, hydronephrosis), and patients with genetic syndromes were excluded.

The sample size was calculated to detect an absolute difference of 20% in the infection rate between newborns exposed or not to ranitidine treatment, with α = 5% and 90% power. We hypothesised that a neonate exposed to ranitidine would be more likely to be infected (30% and 10% of patients exposed or not to ranitidine, respectively) [7]. A sample size of 300 newborns was required and evaluated, of which 115 were exposed to ranitidine and 185 were not. None of them were excluded.

### Outcomes

The primary outcome analysed in the study was the rate of infections in preterm infants exposed or not to treatment with ranitidine. Secondary outcomes were the occurrence of NEC (Bell stage >II), mortality, and hospital stay.

The Brazilian Ministry of Health criteria [13] were used to define nosocomial infection and its types. Therefore, nosocomial infection was defined as a late onset infection starting after 48 h of life. Based on the site of infection, it was further classified as follows: a) pneumonia was determined by clinical signs, such as apnoea, tachypnoea, grunting, bradycardia or tachycardia, wheezing or snoring associated with radiological findings with suggestive signals of pulmonary involvement by infectious agents (persistent infiltrate, consolidation and cavitation) and abnormal laboratory tests; b) meningitis was defined by the cerebrospinal fluid of the microorganism isolation and/or the use of antimicrobial therapy for meningitis by the assistant doctor; c) urinary tract infection (UTI) was defined by the presence of signs and symptoms suggestive of infection associated with positive urine culture; and d) late onset sepsis was considered when there were suggestive signs of infection and a positive blood culture for microorganisms not colonizing the skin. Presumed late onset sepsis was defined by the presence of suggestive symptoms of infection associated with altered laboratory tests (white blood count increase with young neutrophils and positive PCR) and negative blood culture. NEC and Bell stage were decided on the basis of standardised clinical and radiologic criteria [14, 15].

### Data collection

Research assistants collected data from the medical records of newborns, using a pre-defined form, regarding: personal information of the mother; background of the obstetrician; gestational age (GA); birth weight and height; Apgar score; occurrence of infections and/or NEC; presence and duration of intensive care invasive procedures (mechanical ventilation (MV), central catheter peripherally inserted (CCPI), umbilical catheter (UC), parenteral nutrition (PN), orogastric tube (OT); indications, timing and dosage of ranitidine treatment; antibiotic therapy; use of corticosteroid; results of laboratory tests; age in days to discharge or death.

### Data analysis

Categorical variables were described using frequencies and percentages. Pearson’s Chi-square or Fisher Exact Tests were used to compare the categorical variables association. The normal distribution of the scores was verified using the Kolmogorov-Smirnov test. The T-Student or Mann-Whitney tests were used to assess any differences in the study variables between the groups, respecting the distribution symmetry. We calculated the relative risk (RR) and 95% confidence interval (CI). We controlled for possible confounding variables using a backwards stepwise modelling, retaining variables that had a *p*-value smaller than 0.05. Analyses were performed using SPSS version 20.0 (SPSS Inc., Chicago, IL USA) and STATA 12.0 (STATA Corp., College Station, TX, USA).

## Results

A total of 300 neonates admitted to the NICU were enrolled in the study. Of these, 115 (38.3%) used ranitidine, mostly due to episodes of regurgitation, and 60 (52.2%) and 29 (25.2%) due to prophylaxis or treatment of stress ulcer, respectively. 185 neonates represent the control group of patients not exposed to ranitidine. The main demographic and clinical characteristics of both groups were similar, as shown in Table 1. However, neonates, who received ranitidine, were more likely to undergo more interventions and procedures, such as mechanical ventilation [Mean (SD), 5.5 (7.2) vs 2.0 (3.3), *p* < 0.001], umbilical catheter [2.8 (2.7) vs 1.9 (2.6), *p* = 0.007], central catheter peripherally inserted [Mean (SD), 7.2 (8.2) vs 4.0 (6.0), *p* < 0.001], and parenteral nutrition [Mean (SD), 5.9 (5.4) vs 3.0 (4.3)] than neonates who did not receive ranitidine. Additionally, we explored whether the long-term use of these devices was associated with nosocomial infection in neonates and we observed that the use duration of these devices was not associated with a risk of infection (Table 2). Although there is no association between device duration and infection, we performed a multivariate analysis to control for confounding variables because of its clinical relevance and we found that ranitidine was independently associated with a risk of infection (Additional file 1: Table S1).

**Table 1:** Association of maternal and neonatal characteristics in patients who used or did not use ranitidine in a Brazilian NICU, 2014–2015

Variable	Not exposed to Ranitidine	Exposed to Ranitidine	*p*-value
	*n* = 185	*n* = 115	
Maternal and prenatal care information			
Age mother, mean (SD)	25.0 (7.2)	24.5 (6.8)	0.53^a^
> 6 visits prenatal care, n (%)	56 (30.3)	34 (29.6)	0.89^b^
Hypertension, n (%)	16 (8.6)	13 (11.3)	0.45^b^
Diabetes Mellitus, n (%)	1 (0.5)	1 (0.9)	0.73^b^
Gestational diabetes, n (%)	1 (0.5)	1 (0.9)	0.73^b^
Caesarean section delivery, n (%)	95 (51.4)	47 (40.9)	0.08^b^
Characteristics of the neonates			
Male, n (%)	88 (47.6)	60 (52.2)	0.44^b^
Twins, n (%)	31 (16.8)	19 (16.5)	0.95^b^
Gestational age, median (IQR)	32 (31–34)	32 (30–33)	0.26^c^
Birth weight (g), median (IQR)	1474 (1163–1863)	1410 (1150–1810)	0.29^c^
Apgar score at 1 min, median (IQR)	7 (5–8)	7 (5–8)	0.24^c^
Apgar score at 5 min, median (IQR)	9 (8–9)	8 (8–9)	0.16^c^
Devices used by the neonate			
Duration of mechanical ventilation (d), mean (SD)	2.0 (3.3)	5.5 (7.2)	<0.001^a^
Duration of umbilical catheter (d), mean (SD)	1.9 (2.6)	2.8 (2.7)	0.007^a^
Duration of central catheter peripherally inserted (d), mean (SD)	4.0 (6.0)	7.2 (8.2)	<0.001^a^
Duration of orogastric tube (d), mean (SD)	19.9 (15.3)	11.9 (9.3)	<0.001^a^
Duration of parenteral nutrition (d), mean (SD)	3.0 (4.3)	5.9 (5.4)	<0.001^a^

*d* days, *SD* standard deviation, *IQR* interquartile range

^a^T-Student test

^b^Chi-Square test

^c^Mann-Whitney U test

**Table 2:** Association of the use of devices between patients who developed nosocomial infection and those who did not in a Brazilian NICU, 2014–2015

Variable	Infection		*p*-value^a^
	Yes	No	
Duration of mechanical ventilation (d), mean (SD)	3.6 (5.3)	3.3 (5.4)	0.60
Duration of umbilical catheter (d), mean (SD)	2.5 (2.5)	2.2 (2.7)	0.46
Duration of central catheter peripherally inserted (d), mean (SD)	5.4 (7.1)	5.2 (7.1)	0.82
Duration of orogastric tube (d), mean (SD)	16.7 (13.0)	16.7 (14.0)	0.96
Duration of parenteral nutrition (d), mean (SD)	4.8 (5.1)	3.8 (4.7)	0.09

*d* days, *SD* standard deviation

^a^T-Student test

Neonates who received ranitidine were more likely to have nosocomial infection (RR: 1.6, 95% CI = 1.1–2.2, *p* = 0.006), confirmed sepsis (RR: 3.4, 95%CI: 1.4–8.2, *p* = 0.003), and pneumonia (RR: 2.6, 95%CI: 1.2–5.4, *p* = 0.01) (Table 3). The median (IQR) between the first dose of ranitidine and infection outcome was 6 (3–8) days. The most used route of administration was intravenous (104, 90,5%), and the mean (SD) dose was 1.1 (0.33) mg/kg/day. Only 11 (9,5%) used ranitidine by oral administration and the mean (SD) dose was 3.75 (0.9) mg/kg/day.

**Table 3:** Nosocomial infection prevalence in premature neonates admitted to a Brazilian NICU in 2014–2015

Variable, description	Not exposed to Ranitidine	Exposed to Ranitidine	*p*-value
	*n* = 185	*n* = 115	
Overall infection, n (%)	49 (26.5)	48 (41.7)	0.006^a^
Sepsis confirmed, n (%)	7 (3.8)	15 (13.0)	0.003^a^
Pneumonia, n (%)	10 (5.4)	16 (14.0)	0.01^a^
Urinary tract infection, n (%)	0 (0.0)	3 (2.6)	0.03^b^
Fungal infection, n (%)	6 (3.2)	3 (2.6)	0.74^b^

^a^Chi-Square test

^b^Fisher’s exact test

Twenty-one (7%) cases of NEC were reported in the patient groups during the study period and ranitidine use was not significantly associated with necrotising enterocolitis (RR = 1.4, 95%IC: 0.6–3.3, *p* = 0.36).

The mortality rate was significantly higher in neonates who received ranitidine (RR 3.9, 95%CI: 2.1–7.3, *p* < 0.001). Furthermore, hospitalisation was longer in those exposed to ranitidine (Median (IQR), 36.0 (19.0–59.0) vs 24.0 (14.5–38.0), *p* < 0.001).

## Discussion

Despite a lack of sufficient evidence demonstrating the safety and efficacy of IGAS use by preterm newborns [10], these drugs have been widely prescribed in an off-label manner to treat prophylaxis or therapy of stress ulcers and gastroesophageal reflux disease (GERD) in NICU. The use of these drugs and its consequences for preterm newborns have raised important questions. However, the majority of the studies evaluating its safety have been performed in developed countries. We performed a retrospective cohort study in order to compare the rates of hospital infections and NEC between preterm newborns exposed or not to ranitidine treatment, admitted to a NICU in a poor area of Northeastern Brazil. In accordance with studies from developed countries, our results showed an association between ranitidine use and an increased risk of infection [6–8, 16, 17] and mortality [7]. However, there was no significant association between the use of ranitidine and NEC.

Gastric acid secretion is one of the main non-immune defenses of newborns against invading microorganisms [8]. According to some studies, increasing the pH of gastric secretion would result in the overgrowth of microorganisms and in predisposition to infection and NEC [18–21]. Sustained inhibition of gastric acid secretion alters the bacterial ecology favouring gastric colonisation by enteric bacteria and may facilitate microbial translocation across barrier due to decreased neutrophil activity [22, 23]. In fact, the use of ranitidine was reported to increase gastric pH within 30 min of administration [24, 25] and its effects not only restrict gastric secretion, but also activate H_2_ receptors, modelling the immune response, especially in the production of inflammatory cytokines, and reducing infection control [26–28].

In this study, the presence of GERD was the main indication for the use of ranitidine, followed by prophylaxis, or therapy of stress ulcers. Our study showed that children receiving H2-blocker therapy were more likely to exhibit irritability, sleepiness, and other non-specific symptoms, leading to the false interpretation of GERD persistence and consequently leading to an increase of the drug dosage or the duration of treatment [25]. However, our findings showed that the mean dose of ranitidine administered to newborns is in agreement with other studies (range 1–5 mg/Kg/day) (16), although some studies recommend an intravenous dose of 0.5 mg/kg/day for preterm newborns presenting regular renal function [25, 29]. Furthermore, we found that the time of ranitidine use was not associated with an increase in the infection rate. In this study, the time between the first dose of ranitidine and infection outcome was only 6 days, whereas a study conducted in Croatia reported 18 days [17]. Despite these differences, there is currently no consensus with regard to the safe dose and time usage of IGAS therapy for newborns and further studies are needed.

Some studies have shown that the prolonged use of neonatal intensive care devices by neonates and infants, such as mechanical ventilation, central catheter peripherally inserted and parenteral nutrition dispositive, predispose to infections [30–34]. However, in this study, we have found that these devices were used on newborns receiving ranitidine for longer periods of time. After controlling for confounding variables, we found that ranitidine was independently associated with a risk of infection. Indeed, similar findings were reported by Yildizdas et al. [35].

Although some studies have found an association between the use of ranitidine and an increase in NEC [7, 9], this association was not observed in this study. A possible explanation is that those researchers studied very low birth weight newborns, whereas our population was composed of neonates with a larger variation of birth weight. Therefore, it is possible that the effects of ranitidine on quantitative and qualitative changes of the intestinal microflora composition may be causing NEC in very low birth weight newborns.

In this study, we observed an increase in the hospitalisation time and mortality rate amongst newborns receiving ranitidine. This is consistent with a multi-centre study observing the relationship between ranitidine and unfavourable outcomes [7].

This was a retrospective cohort study based on medical reports and some information were not recorded, such as data on timing, volume and type of enteral feedings, rods count and blood cultures. In addition, at NSLM, the search of fungus in urine to investigate fungal infections in neonates frequently results in no clinical and laboratory improvement after antibiotic use. Despite these limitations, our results are consistent.

## Conclusions

In conclusion, ranitidine use was associated with an increased risk of infections and mortality in preterm newborns, but not with NEC. The use of ranitidine in neonates must be further evaluated and used in specific situations. In addition, more studies are needed to increase the information about the use of IGAS in newborns to support the decision of the regulatory agencies authorising and adjusting its use in neonatology.

## Additional file

Additional file 1: Table S1. Multivariate analysis to control for confounding variables. (DOCX 22 kb)

## Abbreviations

CCPI: Central catheter peripherally inserted
GERD: Gastroesophageal reflux disease
IGAS: Inhibitors of gastric acid secretion
MV: Mechanical ventilation
NEC: Necrotising enterocolitis
NICU: Neonatal intensive care units
NSLM: *Nossa Senhora de Lourdes* Maternity
OT: Orogastric tube
PN: Parenteral nutrition
UC: Umbilical catheter
UTI: Urinary tract infection
