# Supplementary material for: Use of ranitidine is associated with infections in newborns hospitalized in a neonatal intensive care unit: a cohort study
Source: BMC Infect Dis. 2017 May 30;17:375. doi: 10.1186/s12879-017-2482-x (PMC5450121; doi:10.1186/s12879-017-2482-x)
Supplement: Additional file 1: Table S1. — Multivariate analysis to control for confounding variables. (DOCX 22 kb) [file 12879_2017_2482_MOESM1_ESM.docx]

**Supplement**

**Table 1. Multivariate analysis to control for confounding variables.**

| **Variables in the Equation** | | | | | | | | | |
| --- | --- | --- | --- | --- | --- | --- | --- | --- | --- |
|  | | B | S.E. | Wald | df | Sig. | Exp(B) | 95% C.I. for EXP(B) | |
|  |  |  |  |  |  |  |  | Lower | Upper |
| Step 1^a^ | Ranitidine(1) | 0.832 | 0.320 | 6.765 | 1 | 0.009 | 2.298 | 1.228 | 4.301 |
|  | Mechanical ventilation (d) | -0.038 | 0.034 | 1.230 | 1 | 0.267 | 0.962 | 0.900 | 1.030 |
|  | Umbilical catheter (d) | -0.013 | 0.055 | 0.058 | 1 | 0.810 | 0.987 | 0.885 | 1.100 |
|  | Central catheter peripherally (d) | -0.027 | 0.027 | 0.972 | 1 | 0.324 | 0.973 | 0.923 | 1.027 |
|  | Orogastric tube (d) | 0.012 | 0.012 | 0.970 | 1 | 0.325 | 1.012 | 0.988 | 1.036 |
|  | Parenteral nutrition (d) | 0.051 | 0.036 | 2.016 | 1 | 0.156 | 1.053 | 0.981 | 1.130 |
|  | Constant | -1.209 | 0.279 | 18.821 | 1 | 0.000 | 0.298 |  |  |
| Step 2^a^ | Ranitidine(1) | 0.818 | 0.315 | 6.769 | 1 | 0.009 | 2.267 | 1.224 | 4.199 |
|  | Mechanical ventilation (d) | -0.040 | 0.033 | 1.457 | 1 | 0.227 | 0.961 | 0.900 | 1.025 |
|  | Central catheter peripherally (d) | -0.025 | 0.026 | 0.916 | 1 | 0.339 | 0.975 | 0.926 | 1.027 |
|  | Orogastric tube (d) | 0.011 | 0.012 | 0.915 | 1 | 0.339 | 1.011 | 0.988 | 1.034 |
|  | Parenteral nutrition (d) | 0.050 | 0.036 | 1.962 | 1 | 0.161 | 1.051 | 0.980 | 1.127 |
|  | Constant | -1.217 | 0.277 | 19.316 | 1 | 0.000 | 0.296 |  |  |
| Step 3^a^ | Ranitidine(1) | 0.690 | 0.282 | 6.011 | 1 | 0.014 | 1.994 | 1.149 | 3.463 |
|  | Mechanical ventilation (d) | -0.042 | 0.033 | 1.572 | 1 | 0.210 | 0.959 | 0.898 | 1.024 |
|  | Central catheter peripherally (d) | -0.017 | 0.025 | 0.466 | 1 | 0.495 | 0.983 | 0.936 | 1.033 |
|  | Parenteral nutrition (d) | 0.055 | 0.035 | 2.428 | 1 | 0.119 | 1.057 | 0.986 | 1.133 |
|  | Constant | -1.039 | 0.201 | 26.827 | 1 | 0.000 | 0.354 |  |  |
| Step 4^a^ | Ranitidine(1) | 0.698 | 0.281 | 6.175 | 1 | 0.013 | 2.010 | 1.159 | 3.486 |
|  | Mechanical ventilation (d) | -0.049 | 0.031 | 2.463 | 1 | 0.117 | 0.952 | 0.895 | 1.012 |
|  | Parenteral nutrition (d) | 0.046 | 0.032 | 1.995 | 1 | 0.158 | 1.047 | 0.982 | 1.116 |
|  | Constant | -1.068 | 0.197 | 29.493 | 1 | 0.000 | 0.344 |  |  |
| Step 5^a^ | Ranitidine(1) | 0.759 | 0.278 | 7.474 | 1 | 0.006 | 2.136 | 1.240 | 3.681 |
|  | Mechanical ventilation (d) | -0.028 | 0.027 | 1.062 | 1 | 0.303 | 0.973 | 0.923 | 1.025 |
|  | Constant | -0.974 | 0.183 | 28.331 | 1 | 0.000 | 0.378 |  |  |
| Step 6^a^ | Ranitidine(1) | 0.669 | 0.264 | 6.445 | 1 | 0.011 | 1.952 | 1.165 | 3.273 |
|  | Constant | -1.028 | 0.176 | 34.247 | 1 | 0.000 | 0.358 |  |  |

(d) = duration in days
